# Are Selenium Status and Inflammation Linked to Cardiometabolic Risk Factors in Inborn Errors of Immunity?

**DOI:** 10.3390/nu18183030

**Published:** 2026-09-16

**Authors:** Rosana Gomes de Torres Rossi, Lucila Pereira, Fabíola Isabel Suano-Souza, Itana Gomes Alves Andrade, Rogério do Prado, Carolina Sanchez Aranda, Agatha Nogueira Previdelli, Roseli Oselka Saccardo Sarni, Maria Teresa Terreri

**Affiliations:** 1 Division of Allergy, Clinical Immunology and Rheumatology, Department of Pediatrics, Universidade Federal de São Paulo (UNIFESP), São Paulo 04025002, Brazil; pereiralucila@hotmail.com (L.P.); suano.souza@unifesp.br (F.I.S.-S.); itanaandrade@yahoo.com.br (I.G.A.A.); carolinaaranda@yahoo.com.br (C.S.A.); rssarni@gmail.com (R.O.S.S.); teterreri@terra.com.br (M.T.T.); 2 Faculdade de Ciências Médicas da Santa Casa de São Paulo (FCMSCSP), São Paulo 01225010, Brazil; 3 Centro Universitário FMABC, Santo André 09060650, Brazil; rogeriodopadro@yahoo.com.br; 4 Facultad de Ciencias de la Salud, Universidad Autónoma de Chile, Providencia, Santiago 7500912, Chile; agatha.usp@gmail.com

**Keywords:** plasma selenium status, ataxia–telangiectasia, common variable immunodeficiency, juvenile systemic lupus erythematosus, oxidative stress, immunity, nutritional status

## Abstract

Background/Objective: Patients with Inborn Errors of Immunity (IEI) are at increased cardiometabolic risk due to inflammation, immune dysfunction, and oxidative stress. Given selenium’s essential role in antioxidant defense, this study evaluated plasma selenium concentration in individuals with IEI and its associations with inflammatory and metabolic biomarkers and body mass index (BMI). Methods: A controlled cross-sectional study involved 85 patients, 22 with ataxia–telangiectasia (A-T), 32 with common variable immunodeficiency (CVI) and 31 with juvenile systemic lupus erythematosus (jSLE). The control group comprised 72 age- and sex-matched healthy individuals. Anthropometric, inflammatory, metabolic, and oxidative stress parameters were assessed, including BMI, ultrasensitive C-reactive protein (usCRP), lipid profile, fasting glucose, insulin, plasma Se concentrations, erythrocyte glutathione peroxidase (GPx) activity, and malondialdehyde levels. Results: Selenium concentrations below the reference value (46 µg/L) were observed in all groups, A-T (41%), CVI (50%), jSLE (48%) and the control group (33%). Low weight was more frequent in the A-T group (41%), whereas excess weight predominated in the CVI (47%) and jSLE (19%) groups. Multiple linear regression showed lower selenium concentrations in the jSLE (β = −10.1 ug/L) and CVI (β = −12.8 µg) groups compared with the controls (*p* < 0.05). Selenium concentrations were associated with age (β = 0.3 µg/L) and usCRP (*p* < 0.05). The GPx values were lower among all patient groups (jSLE: β = 3228 U/L, A-T: β = −2030 U/L, and CVI: β = −2060 U/L) (*p* < 0.05) compared with the controls. The GPx was also influenced by age (β = 56 U/L; *p* = 0.010). Conclusion: The present findings suggest alterations in the endogenous antioxidant defense system in patients with IEI, characterized by reduced erythrocyte GPx activity and lower plasma selenium concentrations. The inverse association between selenium and usCRP concentrations suggests a potential link between selenium status and systemic inflammation. However, these findings should be interpreted cautiously, as inflammation may influence circulating selenium concentrations, and the cross-sectional design does not establish causal inferences. Further longitudinal studies are warranted to clarify the relationship between selenium status, inflammation, and antioxidant defense in IEI.

## 1. Introduction

Selenium is an essential micronutrient for human health, playing a fundamental role in maintaining immunological and antioxidant homeostasis [1]. Selenium deficiency is associated with various health risks, including impaired immune function, exacerbated oxidative stress, and increased susceptibility to chronic diseases. Prasad et al. (2024) emphasized that low plasma selenium may impair selenoprotein activity, particularly glutathione peroxidases, compromising antioxidant defenses and increasing susceptibility to oxidative stress and cardiomyopathy [2].

The relationship between selenium status and health outcomes follows a U-shaped pattern where deficient and excessive selenium concentrations are associated with increased risk of adverse outcomes [3]. However, low plasma selenium concentrations should be interpreted cautiously, as they do not necessarily indicate that selenium supplementation would confer a beneficial effect.

GPx is part of the antioxidant system of all cells using oxidative metabolism and plays an essential role in cellular protection by eliminating hydroperoxides and other reactive species. Erythrocyte GPx activity is considered an important functional marker for assessing the antioxidant properties of selenium [4].

In this context, the role of selenium becomes particularly relevant in diseases characterized by oxidative stress, such as patients with Inborn Errors of Immunity (IEI), who, along with other complications, present elevated cardiometabolic risk due to mechanisms involving chronic inflammation, immune dysfunction, and oxidative stress. These factors contribute to the development of atherosclerosis and insulin resistance [5].

Findings from a recent review highlighted the scarcity of studies evaluating selenium concentrations and the potential benefits of supplementation in patients with autoimmune diseases. Although the authors suggest that increased selenium intake may help reduce complications, including atherosclerosis, in patients with systemic lupus erythematosus (SLE), these findings warrant further investigation, as, as previously mentioned, the relationship between selenium status and health outcomes may follow a U-shaped pattern [6].

Three controlled cross-sectional studies conducted by our research group assessed the plasma selenium status of patients with IEI. The first study evaluated patients with ataxia–telangiectasia (A-T), finding inadequate selenium concentrations in 41% of individuals, which were significantly associated with increased levels of oxidative stress biomarkers and reduced GPx activity [7]. The second study evaluated patients with common variable immunodeficiency (CVI) and demonstrated lower selenium concentrations and GPx activity in patients compared with the healthy controls, suggesting a higher risk of oxidative stress and cardiovascular disease in these patients [8].

Lastly, the third study conducted with children and adolescents with juvenile SLE (jSLE) revealed that approximately 50% of the patients presented plasma selenium concentrations below the reference values, accompanied by a significantly higher prevalence of inadequate GPx activity compared with the healthy controls. Moreover, it was found that there was an independent inverse association between selenium concentrations and LDL cholesterol in both groups, suggesting a protective role of this micronutrient in the development of dyslipidemia in the patients and controls [9].

Given these findings and the limited evidence regarding selenium status in different types of IEI, the present study aims to compare plasma selenium status among individuals with IEI and healthy controls, such as: inflammatory markers, glucose and lipid metabolism, and body mass index, all of which are recognized factors linked to cardiometabolic risk.

## 2. Materials and Methods

This single-center, cross-sectional, observational, and controlled study evaluated three groups of patients with IEI: A-T group (*n* = 22); CVI group (*n* = 32); and jSLE group (*n* = 31). Patients were invited to participate in the study during their medical appointments at the outpatient clinic of Hospital São Paulo, affiliated with the Federal University of São Paulo. The control group consisted of 72 healthy volunteers matched for sex and age, recruited from a Primary Health Care Service. The control group was designed separately for each disease group, ensuring there was no overlap of individuals [7,8,9].

### 2.1. Inclusion and Exclusion Criteria

Patients with A-T and CVI, of all age groups, were included in the study. They were classified based on the 2019 International Union of Immunological Societies (IUIS) criteria [10]. The jSLE group comprised only female individuals between 10 and 18 years old. Disease classification followed the criteria established by the Systemic Lupus International Collaborating Clinics (SLICC), published in 2012 [11]. Disease activity was assessed using the Systemic Lupus Erythematosus Disease Activity Index 2000 (SLEDAI-2K) [12].

Individuals with acute or chronic infections clinically identified within the 15 days prior to laboratory testing were excluded. For the jSLE group, patients receiving pulse therapy of methylprednisolone or cyclophosphamide at the time of blood collection were also excluded [9]. For the CVI group, the exclusion criteria included acute infection and corticosteroid use at the time of laboratory sample collection or during the preceding 3 months [8].

The study was conducted according to the principles expressed in the Declaration of Helsinki and was approved by the Research Ethics Committee of the Federal University of São Paulo. All participants signed consent forms and/or assent forms. The consent of participants younger than 18 years old was obtained from their parents or legal guardians.

### 2.2. Data Collection

Demographic, clinical, laboratory, and disease-specific treatment data were collected from patients’ medical records.

### 2.3. Anthropometric Assessment

Weight and height measurements were taken by a nutritionist according to the guidelines of the World Health Organization (WHO) [13]. Patients unable to remain standing had their weight measured in a wheelchair using a scale designed specifically for wheelchair users (Electronic Scale for Wheelchair Users—Micheletti—capacity 500 kg—Serial: 2161058). Recumbent height was measured with the patient lying on a flat, firm surface, using an inextensible tape, graduated in millimeters.

Among children and adolescents, pubertal staging was assessed by self-evaluation [14] according to the criteria of Marshall & Tanner [15]. Anthropometric classification included the calculation of body mass index-for-age (BMI/A) and height-for-age (H/A), both expressed as Z-scores, using De Onis as a reference for children and adolescents [16]. For adults, BMI classification followed the WHO guidelines [13]. Additionally, the BMI values were also analyzed as continuous variables.

Waist circumference, measured at the midpoint between the lowest rib (tenth) and the iliac crest, was used to calculate the waist-to-height ratio (WHR). A WHR value higher than 0.5 indicated an increased amount of abdominal and/or visceral fat, which may increase the risk of developing cardiometabolic conditions [17].

### 2.4. Biochemical Assessment

The blood sample was collected during the first medical appointment, provided that the patient had undergone a 12 h fasting period. If the patient had not fasted for 12 h, they were invited to return on another day.

A blood sample (15 mL) was collected from each individual via peripheral venipuncture, and the samples were placed in tubes with anticoagulant (ethylenediaminetetraacetic acid—EDTA). All biochemical analyses were performed in the same laboratory, under the same conditions, using standard methods and good clinical laboratory practices. More details can be found in previous publications [7,8,9].

### 2.5. Selenium Nutritional Status

Plasma selenium concentration was determined by graphite furnace atomic absorption spectrophotometry, with a detection limit of 1.0 µg/L and linearity of up to 400.0 µg/L; the coefficient of variation was 0.8%, and values above 46 µg/L were considered desirable. Total erythrocyte GPx activity was determined in whole blood according to Paglia and Valentine (RANDOX, Crumlin, County Antrim, UK) [18], with desirable values above 4171 U/L. The method is linear up to a concentration of 925 U/L. Sensitivity was 75 U/L, and the coefficient of variation was 3.4%.

### 2.6. Lipid and Glucose Metabolism

The lipid profile, including triglycerides (TG), total cholesterol (TC), and HDL-c, was determined using enzymatic-colorimetric methods. Low-density lipoprotein cholesterol (LDL-c) and very-low-density lipoprotein (VLDL-c) concentrations were estimated according to the Friedewald et al. equation [19]. They were classified based on the cut-off points recommended by the American Academy of Pediatrics [20] and by the American College of Cardiology/American Heart Association (ACC/AHA) [21]. Dyslipidemia was defined as the presence of at least one biochemical parameter exceeding the established range: TC > 170 mg/dL for children/adolescents and >200 mg/dL for adults and/or LDL-c > 110 mg/dL for children/adolescents and >129 mg/dL for adults and/or triglycerides >100 mg/dL for children/adolescents and >150 mg/dL for adults and/or HDL-c < 35 mg/dL for children/adolescents, <40 mg/dL for women and <50 mg/dL for men.

Glucose plasma concentrations were determined using the GLUC3/Roche kit (Indianapolis, IN, USA) based on the reference enzymatic method with hexokinase. Insulin concentrations were quantified using the Elecsys Insulin/Roche kit (Indianapolis, IN, USA), based on electrochemiluminescence. HOMA-IR (Homeostasis Model Assessment of Insulin Resistance) was calculated from fasting glucose and insulin concentrations according to the following formula [22]:

HOMA-IR = fasting glucose (mmol/L) × fasting insulin (μU/mL)/22.5.

### 2.7. Malondialdehyde and Ultrasensitive C-Reactive Protein

Serum malondialdehyde (MDA) concentration was determined using a colorimetric method (in-house methodology), with the desirable value defined as ≤4.8 nmol/mL. Ultrasensitive C-reactive protein (usCRP) was quantified by a turbidimetric-immunologic method using a Roche assay (Mannheim, Baden-Württemberg, Germany), with an analytical range of 1.0 to 200 mg/L and a coefficient of variation of 1.3%. Further methodological details are provided in our previously published article [8].

### 2.8. Statistical Analysis

Categorical variables were expressed as absolute frequencies and percentages and compared using the Pearson chi-square or Fisher’s exact test, as appropriate. The distribution of continuous variables was assessed for normality using kurtosis values, visual inspection of histograms, and the Kolmogorov–Smirnov test. Variables with parametric distribution were presented as mean ± standard deviation and compared using Student’s *t*-test and the Pearson correlation coefficient. Non-parametric variables were expressed as median and interquartile range and compared using the Mann–Whitney test and Spearman rank correlation coefficient. Multiple linear regression analyses were performed with categorical variables (groups) entered into the models as dummy variables. Variables associated with selenium and GPx concentrations in the binary analysis were included in the multiple regression.

Statistical analysis was performed using SPSS 29.0 (IBM corp, Armonk, NY, USA). The significance level was set at 5% (*p* < 0.05).

## 3. Results

This study included 85 individuals diagnosed with Inborn Errors of Immunity (IEI), comprising 22 patients with ataxia–telangiectasia (A-T), 32 with common variable immunodeficiency (CVI) and 31 with juvenile systemic lupus erythematosus (jSLE). Table 1 shows the demographic data of these three groups: A-T, CVI, and jSLE.

Dyslipidemia occurred in 16/22 (72.7%) patients of the A-T group, in 23/32 (71.9%) patients of the CVI group, and in 15/31 (48.4%) patients of the jSLE group.

All patients with CVI and 12/22 (54.5%) patients with A-T were receiving intravenous immunoglobulin replacement therapy. In the CVI group, 14/32 (43.8%) patients had chronic lung disease and were on continuous prophylactic antibiotics, while 5/32 (15.6%) patients had chronic diarrhea. In the jSLE group, 12/31 (38.7%) patients had SLEDAI-2K ≥ 4, and 11/31 (35.5%) patients had kidney involvement. The cumulative corticosteroid dose was 381.2 mg. Twenty-eight of the 31 patients (90.3%) were on hydroxychloroquine, 23/31 (74.2%) patients were on azathioprine, and 7/31 (22.6%) patients were on mycophenolate mofetil. 

Plasma selenium concentrations below the reference value (46 µg/L) were found in 9/22 (40.9%) patients in the A-T group, 16/32 (50%) patients in the CVI group, 15/31 (48.4%) patients in the jSLE group, and 24/72 (33.3%) patients in the control group. Table 2 compares the anthropometric and laboratory variables among the A-T, CVI, jSLE, and control groups. The groups differed in age and sex due to the nature of their diseases. BMI was lower in the A-T group, and usCRP levels were higher in the CVI group. Total cholesterol (*p* = 0.017), LDL-c (*p* < 0.001), and non-HDL cholesterol (*p* = 0.044) were higher in the A-T group. HOMA-IR was significantly higher (*p* < 0.001), and selenium (*p* = 0.036) and GPx activity (*p* = 0.026) were lower in the jSLE group.

**Figure 1:**
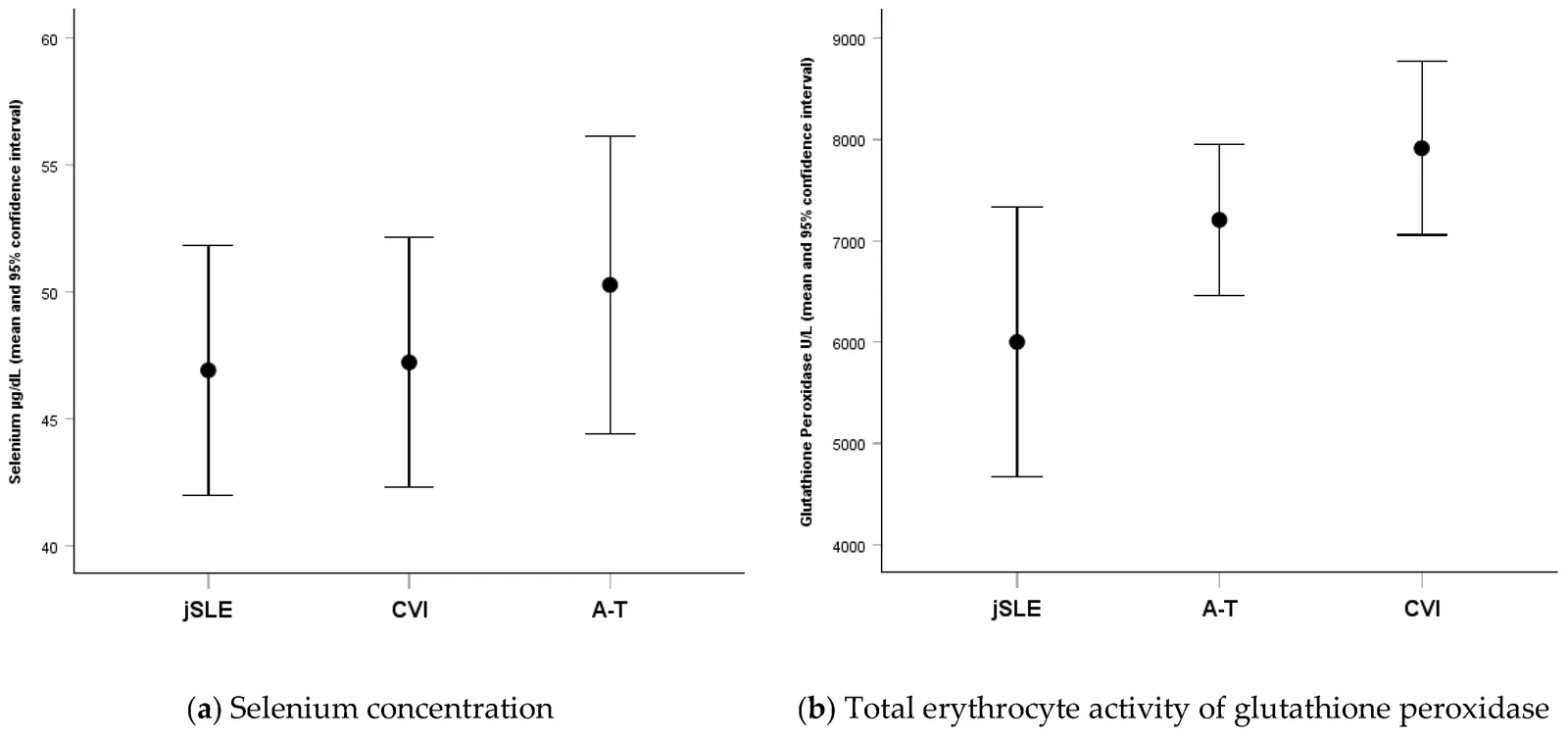
Comparison between selenium concentration and total erythrocyte activity of glutathione peroxidase among the ataxia–telangiectasia, common variable immunodeficiency and juvenile systemic lupus erythematosus groups. Abbreviation: jSLE, juvenile systemic lupus erythematosus; A-T, ataxia–telangiectasia; CVI, common variable immunodeficiency. Significance level of selenium (Student’s *t*-test): jLES vs. A-T *p* = 0.370, jLES vs. A-T *p* = 0.370 and CVI vs. A-T *p* = 0.417. Significance level of glutathione peroxidase (Student’s *t*-test): jLES vs. A-T *p* = 0.113, jLES vs. CVI *p* = 0.017 and A-T vs. CVI *p* = 0.234.

Figure 1 shows the comparison of selenium concentrations and GPx activity among the IEI groups. No significant correlations were found between selenium concentrations and GPx activity values in the A-T (r = −0.024, *p* = 0.914), CVI (r = 0.342, *p* = 0.056) and jSLE (r = −0.144, *p* = 0.439) groups. In the A-T group, selenium concentrations were positively correlated with blood glucose (r = 0.356, *p* = 0.036), whereas GPx activity was inversely correlated with usCRP (rho = −0.361, *p* = 0.025). In the jSLE group, selenium concentrations were inversely correlated with usCRP (rho = −0.399, *p* = 0.026) and LDL-c (r = −0.375, *p* = 0.046). No significant correlations were found between GPx activity and current disease activity, assessed by the Systemic Lupus Erythematosus Disease Activity Index 2000 (SLEDAI-2K) (rho = 0.162, *p* = 0.385, *n* = 31), current corticosteroid dose in mg/kg/day (rho = 0.163, *p* = 0.532, *n* = 17), or cumulative corticosteroid dose in mg/kg (rho = 0.015, *p* = 0.936, *n* = 30). In addition, the current SLEDAI-2K was not significantly correlated with the current corticosteroid dose (rho = 0.033, *p* = 0.901, *n* = 17) or cumulative corticosteroid dose (rho = 0.133, *p* = 0.485, *n* = 30), and the current corticosteroid dose was not significantly correlated with the cumulative corticosteroid dose (rho = 0.029, *p* = 0.911, *n* = 17).

Multiple linear regression showed that the jSLE group (β = −10.1 µg/L; 95% CI −17.8 to −2.5 µg/L; *p* = 0.009) and CVI group (β = −12.8 µg/L; 95% CI −23.2 to −2.3 µg/L; *p* = 0.017) had significantly lower selenium concentrations than the control group. In addition, selenium concentrations were also associated with age (β = 0.32 µg/L; 95% CI 0.10 to 0.54 µg/L; *p* = 0.04) and usCRP (−4.61 mg/L; 95% CI −8.5 to −0.70 µg/L; *p* = 0.021) (Table 3).

The GPx activity was significantly lower in the jSLE group (β = 3228.2; 95% CI 5803.3 to 11,628.4; *p* < 0.001), the A-T group (β = −2030.9; 95% CI −4690.4 to −1765.9; *p* = 0.029), and the CVI group (β = −2060.9; 95% CI −4070.4 to −51.5; *p* = 0.044) compared with the control group. GPx activity was also associated with age (β = 56.1; 95% CI 13.7 to 98.5; *p* = 0.010) (Table 4).

## 4. Discussion

In the present study, plasma selenium concentrations were significantly lower in the jSLE and CVI groups compared to the control group. Additionally, total GPx activity was significantly reduced across all three patient groups (jSLE, A-T, and CVI) compared to the controls. These findings highlight the importance of evaluating plasma selenium status in individuals with IEI, considering the relationship between antioxidant defense and immune dysfunction, with potential implications for disease pathophysiology and progression. To the best of our knowledge, this is the first study to directly compare selenium plasma concentrations across different IEI conditions.

Selenium is known to act as a cofactor for GPx, a key antioxidant enzyme that catalyzes the reduction in lipid hydroperoxides and hydrogen peroxide, protecting cells from oxidative stress [23]. The present study revealed a high prevalence of individuals with selenium concentrations below the established reference range. However, it is important to begin the discussion of the findings by emphasizing that the high prevalence of low plasma selenium concentrations may be partly explained by inflammation-related alterations in selenium metabolism and distribution, as the systemic inflammatory response can contribute to a reduction in circulating selenium concentrations, in addition to increased metabolic demands for this nutrient.

Regarding CVI, a previous study involving 21 patients and 27 healthy controls evaluated antioxidant defense biomarkers and showed lower serum catalase levels in patients, suggesting an impaired antioxidant defense system in response to increased free radical production [24].

Another study demonstrated that individuals with SLE had increased oxidative stress and reduced antioxidant capacity [25]. Low plasma selenium concentrations and reduced GPx activity may contribute to redox imbalance, exacerbating oxidative stress, a key mechanism in SLE pathogenesis and its complications, including cardiovascular disease [26].

A randomized study evaluated the impact of selenium supplementation (200 micrograms/day for 8 weeks) on biomarkers related to inflammation and antioxidant defense in SLE patients. The authors found that the supplementation significantly reduced usCRP, ESR, and malondialdehyde (MDA) levels while increasing GPx activity and total antioxidant capacity (TAC). Clinical manifestations such as arthritis and alopecia also improved after intervention [27]. Nevertheless, supplementation should be approached with caution, as the relationship between selenium status and health outcomes is U-shaped, with both low and excessive selenium levels being associated with adverse outcomes [3]. Therefore, low plasma selenium concentrations should be interpreted cautiously and do not necessarily indicate a need for supplementation.

The high prevalence of cardiovascular complications in individuals with IEI likely reflects the complex interface between immune dysfunction, chronic inflammation, and oxidative stress [28,29]. Although the present study did not assess dietary selenium intake, it is important to mention that a recent population-based study involving 25,801 U.S. participants evaluated the association between selenium intake and the risk of chronic diseases, as well as all-cause mortality. Dietary selenium intake was categorized into quintiles (Q1–Q5). After covariate adjustment, the results showed that participants in the higher quintiles (Q4 and Q5) had a lower risk of cardiovascular disease (OR = 0.97, 95% CI: 0.96–0.99; OR = 0.98, 95% CI: 0.97–1.00, respectively). Additionally, survival curves showed a significant non-linear association between selenium intake and all-cause mortality risk (HR = 0.82, 95% CI: 0.68–0.99), highlighting the importance of this micronutrient [30]. The estimated global prevalence of inadequate selenium intake is 38%, representing approximately 2.8 billion people [31]. However, as the optimal selenium intake and the extent to which dietary selenium influences circulating plasma selenium concentrations have not been clearly established, caution is warranted when interpreting these findings.

The low GPx activity observed across all three patient groups, compared with the healthy controls, may be attributable to multiple pathophysiological mechanisms. One hypothesis is that chronic oxidative stress in these diseases leads to depletion of endogenous antioxidant systems, including GPx, which converts hydrogen peroxide into water to prevent oxidative damage to membranes and cellular DNA [32].

In A-T, defects in the ATM gene impair the oxidative damage response, potentially reducing GPx expression or functional activity, worsening progressive neurodegeneration, and increasing cancer risk [33]. In CVI, chronic immune activation and recurrent infections may promote excessive reactive oxygen species (ROS) production, overwhelming antioxidant defenses such as GPx [34].

In jSLE, immune hyperactivity and chronic systemic inflammation are continuous sources of free radicals, and the literature shows that patients often present with altered antioxidant enzyme levels, including GPx and superoxide dismutase (SOD) [35]. Thus, the reduced GPx activity observed in patients with IEI may contribute to sustained oxidative stress and inflammation, thereby promoting tissue damage and increasing the risk of complications, including early atherosclerosis, mitochondrial dysfunction, and adverse clinical outcomes.

In the jSLE group selenium concentrations were inversely correlated with both usCRP and LDL-c levels. Increased oxidative stress plays a central role in jSLE pathophysiology, leading to abnormal immune system activation, autoantibody production, and other complications. In T cells, mitochondrial dysfunction results in the accumulation of lipid hydroperoxides, exacerbating oxidative stress. In both SLE patients and experimental models, the glutathione depletion (the main intracellular antioxidant) and the redox-dependent mTOR kinase activation have been observed [26].

The independent and inverse association between plasma selenium concentrations and usCRP levels reinforces the role of systemic inflammation in modulating selenium status. This finding is consistent with the previous literature evidence, indicating that selenium is a micronutrient sensitive to inflammatory states, with reduced bioavailability during chronic inflammation, driven by tissue redistribution, increased cellular demand, and altered expression of selenoproteins [36]. Approximately one-third of the healthy controls had plasma selenium concentrations below the reference value, suggesting that suboptimal selenium status may not be specific to individuals with Inborn Errors of Immunity (IEI) and may partly reflect population-level factors, such as dietary patterns and regional selenium availability. Nevertheless, the significantly lower selenium concentrations observed in patients with CVI and jSLE compared with the controls suggest that disease-related factors, including chronic inflammation, altered metabolism, and increased oxidative stress, may further contribute to alterations in selenium status [37,38].

Although the present study, as well as the existing literature, demonstrated an inverse association between plasma selenium concentrations and some inflammatory biomarkers, these findings should be interpreted with caution, as inflammation associated with the underlying diseases (jSLE, A-T, and CVI) may influence plasma selenium concentrations independently of overall selenium stores, potentially limiting its interpretation as a biomarker of selenium status in individuals with inflammatory conditions [39].

In patients with IEI, chronic inflammation, even at subclinical levels, may increase the demand of antioxidant selenoenzymes such as GPx, compromising circulating selenium levels, especially when dietary intake and/or absorption are inadequate. This imbalance between demand and availability may perpetuate a pro-inflammatory and pro-oxidative state, promoting tissue injury, endothelial dysfunction, and increased risk of complications such as atherosclerosis [29]. These findings should be interpreted in the context of the non-linear relationship between selenium status and health outcomes. Although supplementation may be beneficial in individuals with inadequate selenium status, indiscriminate selenium supplementation should be avoided, as excessive selenium exposure may potentially be associated with adverse health outcomes [3,38].

It is important to highlight several key strengths and limitations of the present study. This study has important methodological strengths, such as the inclusion of patients with three distinct IEI diseases and the use of functional and biochemical markers to assess selenium status, including erythrocyte GPx activity. The first limitation is the relatively small sample size in some subgroups, particularly the A-T group, which may have limited statistical power to detect subtle associations; the lack of intra-erythrocyte selenium and other selenoproteins such as selenoprotein P; and heterogeneity in age ranges across IEI groups. Another limitation is the lack of a longitudinal analysis, which would have allowed for the assessment of fluctuations in plasma selenium concentrations over time, as well as the lack of evaluation of dietary selenium intake. It is also important to highlight that certain factors, such as disease activity (SLEDAI-2K), corticosteroid exposure, and specific therapeutic interventions, which may influence selenium status and GPx activity, were not assessed in the present study. The authors highlight that A-T, CVI, and jSLE are clinically and phenotypically distinct disorders, with CVI being particularly heterogeneous. The inclusion of these three groups was not based on phenotypic similarity or the presence of cardiomyopathy, but rather on shared pathophysiological features relevant to the objectives of this study, including immune dysregulation, chronic inflammation, oxidative stress, and impaired antioxidant defense. Although cardiomyopathy is not a typical manifestation of A-T, our study focused on cardiometabolic risk factors, including lipid profile, glucose metabolism, insulin resistance, BMI, and inflammatory markers, rather than on cardiomyopathy itself. Notably, 72.7% of patients with A-T in our cohort presented dyslipidemia. We also recognize the substantial clinical and molecular heterogeneity of CVI. In our cohort, all patients with CVI were receiving intravenous immunoglobulin replacement therapy, while 43.8% had chronic lung disease and 15.6% had chronic diarrhea. However, detailed molecular characterization was not available for all participants, which should be considered when interpreting the findings. As dietary selenium intake was not assessed, it was not possible to determine whether the lower plasma selenium concentrations observed in some IEI groups reflect inadequate dietary intake or are influenced by inflammation-induced redistribution, altered selenium metabolism, or increased physiological requirements associated with chronic disease. Future studies should combine dietary assessment with plasma selenium status and, if possible, selenoprotein biomarkers to better characterize selenium status in individuals with IEI [38]. Given the cross-sectional nature of the present study, the observed associations should be interpreted with caution, as this study design does not allow causal relationships to be established. Although lower selenium concentrations were associated with markers of inflammation and cardiometabolic risk, these findings do not establish whether inadequate selenium status contributes to oxidative stress, inflammation, or cardiometabolic alterations. Conversely, reverse causality should also be considered, as chronic inflammation may influence circulating selenium concentrations through alterations in selenium metabolism, distribution, and utilization. Therefore, the observed relationships should be interpreted cautiously, and longitudinal studies are warranted to clarify the temporal and potential causal relationships between selenium status, inflammation, oxidative stress, and cardiometabolic risk. Finally, another important strength to highlight concerns the study population. The A-T, CVI, and jSLE groups included in the present manuscript are the same patient cohorts previously described [7,8,9]. However, the objectives and analyses of the present study differed from those of the previous publications. While the earlier studies separately investigated selenium-related parameters within each specific IEI group, the present study provides a comparative evaluation of plasma selenium concentrations and erythrocyte GPx activity across the three IEI groups and healthy controls. The between-group comparisons, as well as the analyses examining associations between selenium status, GPx activity, inflammatory and cardiometabolic biomarkers, and the multivariable models presented in this study, were conducted specifically for the present investigation. Thus, although the cohorts have been previously characterized, the comparative analyses and integrated interpretation presented here represent new analyses.

## 5. Conclusions

The lower plasma selenium concentrations observed in patients with jSLE and CVI, together with reduced erythrocyte GPx activity across all three IEI groups, suggest alterations in selenium-related antioxidant defense in these immune-mediated conditions. The inverse association between plasma selenium concentrations and usCRP suggests a potential relationship between systemic inflammation and selenium status. In contrast, selenium concentrations were not significantly associated with glucose metabolism biomarkers or BMI in this population. However, we should highlight the importance of considering inflammatory status when interpreting circulating selenium concentrations in patients with IEI. Clinically, these findings support a more comprehensive assessment of nutritional, inflammatory, and cardiometabolic status in these populations, rather than the isolated interpretation of plasma selenium concentrations. Nevertheless, the present findings do not support routine selenium supplementation, as low plasma selenium may reflect inflammation-related alterations in selenium metabolism and distribution and does not necessarily indicate depleted selenium stores. Also, given the cross-sectional design and the absence of dietary selenium assessment, the present findings do not allow causal relationships to be established or the underlying mechanisms of the observed alterations to be determined. Further longitudinal studies incorporating dietary assessment and additional biomarkers of selenium status are warranted to clarify the temporal and potential causal relationship between selenium status, inflammation, oxidative stress, and cardiometabolic factors in these populations.

## Figures and Tables

**Table 1:** General characteristics of patients according to the three Inborn Errors of Immunity diseases.

Variables	A-T (*n* = 22)	CVI (*n* = 32)	jSLE (*n* = 31)	Control (*n* = 72)
Age, years	12.2 (3.3;27.9) ^1^	36.8 (9.6;61.4) ^1^	16.0 (12;18.2) ^1^	17.1 (3.8;60.2) ^1^
Female, *n* (%)	6 (37.3) ^2^	18 (56.2) ^2^	31 (100) ^2^	54 (75) ^2^
BMI, kg/m^2^				
Underweight, *n* (%)	9 (40.9) ^2^	2 (6.2) ^2^	0 (0.0) ^2^	2 (2.8) ^2^
Normal, *n* (%)	11 (50.0) ^2^	15 (46.9) ^2^	25 (80.6) ^2^	51 (70.8) ^2^
Overweight, *n* (%)	2 (9.1) ^2^	15 (46.9) ^2^	6 (19.4) ^2^	19 (26.4) ^2^
WHR (>0.5), *n* (%)	5 (15.6) ^2^	17 (53.1) ^2^	8 (25.8) ^2^	17 (23.6) ^2^
Disease duration (from diagnosis), years	7.1 (0.7;20.2) ^1^	5.3 (0.5;16.4) ^1^	2.8 (0.8;12.3) ^1^	Not applicable

^1^ Values presented as median (minimum;maximum). ^2^ Values presented as *n* (%). Abbreviations: A-T, ataxia–telangiectasia; CVI, common variable immunodeficiency; jSLE, juvenile systemic lupus erythematosus; BMI, body mass index; WHR, waist-to-height ratio.

**Table 2:** Comparison of BMI and laboratory characteristics among A-T, CVI, jSLE, and control group.

Variables	A-T	CVI	jSLE	Control	*p*-Value
	(*n* = 22)	(*n* = 32)	(*n* = 31)	(*n* = 72)	
BMI (kg/m^2^)	15.7 ± 2.3	24.0 ± 4.2	23.0 ± 4.4	22.3 ± 4.6	<0.001 ^a^
TC (mg/dL)	196.6 ± 44.5	165.7 ± 35.7	168.4 ± 43.1	172.2 ± 40.8	0.037 ^a^
LDL-c (mg/dL)	128.1 ± 44.0	101.2 ± 29.1	78.8 ± 49.7	100.9 ± 32.2	<0.001 ^a^
HDL-c (mg/dL)	50.2 ± 12.5	44.7 ± 13.2	47.1 ± 15.3	52.8 ± 17.6	0.081 ^a^
Triglycerides (mg/dL)	91.1 ± 37.7	95.4 ± 29.5	109.4 ± 53.3	93.0 ± 45.5	0.320 ^a^
Non-HDL-c (mg/dL)	146.3 ± 47.5	121.0 ± 32.5	121.3 ± 41.1	119.4 ± 37.6	0.038 ^a^
Glucose (mg/dL)	89.3 ± 31.5	86.0 ± 9.3	82.6 ± 5.6	88.6 ± 30.0	0.652
**Selenium (µg/dL)**	50.3 ± 13.2	47.2 ± 13.7	46.9 ± 13.4	54.1 ± 14.3	**0.036 ^a^**
**GPx (U/dL)**	7205.9 ± 1686.8	7914.2 ± 2372.6	6000.3 ± 3637.2	8866.8 ± 2404.4	**<0.001 ^a^**
**Malondialdehyde (nmol/mL)**	3.58 ± 0.68	3.54 ± 0.83	3.23 ± 0.66	3.24 ± 0.76	0.098 ^a^
**usCRP (mg/dL)**	1.0 (0.3;9.8)	6.4 (1.2;19.5)	0.68 (0.59;1.65)	0.9 (0.3;2.2)	**<0.001 ^b^**
**Insulin (µU/mL)**	6.2 (2.4;13.5)	6.6 (6.5;10.9)	20.1 (12.6;22.9)	9.7 (6.2;15.4)	**<0.001 ^b^**
**HOMA**	1.27(0.41;3.93)	1.42 (0.55;2.48)	3.98 (2.64;4.62)	1.9 (1.2;3.5)	**<0.001 ^b^**

^a^ *p*-Values were obtained using one-way analysis of variance (ANOVA) for normally distributed variables, which are presented as mean ± standard deviation. ^b^ *p*-Values were obtained using the Kruskal–Wallis test for non-normally distributed variables, which are presented as median (minimum;maximum). Abbreviations: A-T, ataxia–telangiectasia; CVI, common variable immunodeficiency; jLES, juvenile systemic lupus erythematosus; BMI, body mass index; usCRP, ultrasensitive C-reactive Protein; TC, total cholesterol; LDL-c, low-density lipoprotein cholesterol; HDL-c, high-density lipoprotein cholesterol; non-HDL-c, non-high-density lipoprotein cholesterol; HOMA, Homeostasis Model Assessment of Insulin Resistance; GPx, total erythrocyte activity of glutathione peroxidase.

**Table 3:** Multiple linear regression of the factors associated with selenium concentration.

Variables	β-Value	95% CI	*p*-Value
jSLE	−10.10	−17.8 to −2.5	0.009
A-T	−5.01	−14.4 to 4.4	0.297
CVI	−12.8	−23.2 to −2.3	0.017
Age (Years)	0.32	0.10 to 0.54	0.004
BMI (kg/m^2^)	0.05	−0.54 to 0.65	0.861
usCRP (Log10)	−4.61	−8.5 to −0.70	0.021
HOMA	−0.01	−1.21 to 1.19	0.989
MDA	−0.65	−3.56 to 2.24	0.655
Non-HDL-c	0.04	−0.02 to 0.10	0.171

Dependent variable: selenium (µg/L). Reference group = control. Abbreviation: jSLE, juvenile systemic lupus erythematosus; A-T, ataxia–telangiectasia; CVI, common variable immunodeficiency; BMI, body mass index; usCRP, ultrasensitive C-reactive protein; HOMA, Homeostasis Model Assessment of Insulin Resistance; MDA, malondialdehyde; non-HDL-c, non-high-density lipoprotein cholesterol.

**Table 4:** Multiple linear regression of factors associated with total erythrocyte glutathione peroxidase (GPx) activity.

Variables	β-Value	95% CI	*p*-Value
jSLE	3228.2	5803.3 to 11,628.4	<0.001
A-T	−2030.9	−4690.4 to −1765.9	0.029
CVI	−2060.9	−4070.4 to −51.5	0.044
Age (Years)	56.1	13.7 to 98.5	0.010
BMI (kg/m^2^)	−15.2	−130.7 to 100.2	0.795
usCRP (Log10)	−621.6	−1373.1 to 129.9	0.104
HOMA	−47.0	−277.8 to 183.8	0.688
MDA	15.3	−542.1 to 572.6	0.957
Non-HDL-c	5.5	−5.9 to 16.9	0.341

Dependent variable: total erythrocyte activity of glutathione peroxidase (U/L). Reference group = control. Abbreviation: jSLE, juvenile systemic lupus erythematosus; A-T, ataxia–telangiectasia; CVI, common variable immunodeficiency; BMI, body mass index; usCRP, ultrasensitive C-reactive protein; HOMA, Homeostasis Model Assessment of Insulin Resistance; MDA, malondialdehyde; non-HDL-c, non-high-density lipoprotein cholesterol.

## Data Availability

All data generated or analyzed during this study are included in this published article.

## Abbreviations

The following abbreviations are used in this manuscript:

A-T	Ataxia–telangiectasia
TC	Total cholesterol
H	Height
EDTA	Ethylenediaminetetraacetic acid
IEI	Inborn Errors of Immunity
GPx	Glutathione peroxidase
HDL	High-density lipoprotein
HOMA-IR	Homeostasis Model Assessment—Insulin Resistance
A	Age
CVI	Common variable immunodeficiency
BMI	Body mass index
SLE	Systemic lupus erythematosus
jSLE	Juvenile systemic lupus erythematosus
MDA	Malondialdehyde
usCRP	ultrasensitive C-reactive protein
WHR	Waist-to-height ratio
Se	Selenium
SLEDAI-2K	Systemic Lupus Erythematosus Activity Index 2000
SLICC	Systemic Lupus International Collaborating Clinics
SOD	Superoxide dismutase
TG	Triglycerides
VLDL	Very-low-density lipoprotein
WHO	World Health Organization
